# Combining RANK/RANKL and ERBB-2 targeting as a novel strategy in ERBB-2-positive breast carcinomas

**DOI:** 10.1186/s13058-019-1226-9

**Published:** 2019-12-03

**Authors:** Ilianna Zoi, Michalis V. Karamouzis, Evangelia Xingi, Panagiotis Sarantis, Dimitra Thomaidou, Panayiotis Lembessis, Stamatios Theocharis, Athanasios G. Papavassiliou

**Affiliations:** 1 Molecular Oncology Unit, Department of Biological Chemistry, Medical School, National and Kapodistrian University of Athens, 75, M. Asias Street, 11527 Athens, Greece; 2 First Department of Internal Medicine, ‘Laiko’ Hospital, Medical School, National and Kapodistrian University of Athens, 11527 Athens, Greece; 3 Light Microscopy Unit, Hellenic Pasteur Institute, Athens, Greece; 4 Department of Physiology, Medical School, National and Kapodistrian University of Athens, 11527 Athens, Greece; 5 Department of Pathology, Medical School, National and Kapodistrian University of Athens, 11527 Athens, Greece

**Keywords:** Breast cancer, RANK, RANKL, ERBB family, ERBB-2, Dimers, Denosumab, Trastuzumab, Pertuzumab

## Abstract

**Background:**

ERBB-2 is overexpressed in about 20% of breast cancers (BCs), indicating poor prognosis. The receptor activator of nuclear factor-κB (RANK) pathway is implicated in ERBB-2 (+) BC. The purpose of this study was to elucidate the underlying molecular mechanism of this interaction and the beneficial impact of dual targeting of RANK and ERBB-2 pathways.

**Methods:**

We used SKBR3, MCF7, MDA-MB-453, and BT-474 human BC cell lines. We examined RANK and RANKL expression using RT-PCR, Western blot, and immunofluorescence. The evaluation of RANK expression in a cohort of BC patients was performed using immunohistochemistry. The interaction between RANK and ERBB family members was detected using proximity ligation assay (PLA), which enables the visualization of interacting proteins. We used inhibitors of both pathways [trastuzumab (T), pertuzumab (P), denosumab (D)]. NF-κB pathway activation was studied using Western blot. Cell growth and viability was evaluated using XTT, flow cytometry, and clonogenic assay. For cell migration evaluation, scratch assay was performed. Data were analyzed by one-way ANOVA.

**Results:**

Cell lines express RANK and RANKL. RANK immunostaining was also detected in human BC tissue samples. RANK receptor dimerizes with ERBB family members. RANK/ERBB-2 dimer number seems to be associated with ERBB-2 expression (SKBR3, 5.4; BT-474, 8.2; MCF7, 0.7; MDA-MB-453, 0.3). RANK/ERBB-2 dimers were decreased in the presence of the inhibitors D, T, and P, while they were increased after RANKL (R) treatment in SKBR3 (m, 5.4; D, 1.2; T, 1.9; DT, 0.6; TP, 1; DTP, 0.4; R, 11.8) and BT-474 (m, 8.2; D, 3.1; T, 4.3; DT, 0.7; TP, 3.4; DTP, 3.2; R, 11.6). Combination targeting of SKBR3 further decreased NF-κB pathway activation compared to single targeting. In SKBR3, RANKL and ERBB-2 blockage resulted in reduced cell proliferation, increased apoptosis, and lower metastatic potential compared to mock cells (m) and reversed values in RANKL presence. The combination treatment of SKBR3 with D, T, and P had an advantage in functional traits compared to single targeting. Denosumab suppressed NF-κB signaling and diminished proliferation rate in MDA-MB-453 cells. MCF7 did not correspond to inhibitors.

**Conclusions:**

The results indicate a novel physical and molecular association between ERBB-2 and RANK pathways that affects ERBB-2 (+) BC growth. We also present data suggesting that the combination of anti-ERBB-2 agents and RANKL inhibitors have a potential direct anti-tumor effect and should be further tested in certain BC patients.

## Background

According to global statistics, breast cancer (BC) is the most common cancer in the female population [1, 2]. BC is a highly heterogeneous disease, which has been classified into distinct subtypes according to clinicopathologic features and molecular profile [3, 4]. The molecular taxonomy is mandatory for prognosis, diagnosis, and prediction of therapy response [5]. Estrogen receptor-*α* (ER-*α*), progesterone receptor (PR), and epidermal growth factor receptor 2 (ERBB2, HER-2/neu) are the most commonly used biomarkers for BC, and their status determines the main BC subtypes.

ERBB2-positive (ERBB2+) breast carcinomas comprise around 20% of BC cases, are defined by its amplification or overexpression, and indicate poor clinical prognosis [5]. ERBB2 is a member of the ERBB receptor tyrosine kinase (RTK) family, which consists of four members including EGFR, ERBB3, and ERBB4 [6]. Although ERBB2 lacks a ligand-binding domain, it forms homodimers and heterodimers, exhibiting robust signaling activity in ERBB2 (+) BC subset [7]. Over the past decades, powerful ERBB2-targeted agents have been developed. Trastuzumab was the first recombinant antibody against ERBB2 approved for the treatment of ERBB2 (+) BC [8]. Although trastuzumab is remarkably efficient, the percentage of relapsed breast cancer patients is quite worrying, highlighting the need of exploring alternative therapeutic targets in this disease [9]. The developed resistance relies on various mechanisms. Trastuzumab-based combinations are a strategy aiming to overcome the resistance and increase treatment efficiency.

The receptor activator of nuclear factor (NF)-κB, RANK, and its ligand, RANKL, have been recently implicated in BC initiation, progression, and metastasis [10–13]. Particularly, the RANKL/RANK axis has been reported to play a crucial role in progesterone-driven mammary tumorigenesis [10, 13]. The RANK pathway has been also associated with tumor initiation in *BRCA1*-mutation carriers, introducing RANK (+) luminal progenitor cells as the main target population in this BC subtype [14]. Ithimakin et al. demonstrated that RANKL stimulation increased ERBB2 expression in luminal breast cancer stem cells (CSCs), regulating their self-renewal [15]. Probably RANK-mediated IKKα [inhibitor of NF-κB (IκB) kinase subunit α]-NF-κB activation is associated with this regulation [16] and the increase of ERBB2 expression, which further activates NF-κB, generating a positive feedback loop [17].

Interestingly, scientific data have implicated the RANK/RANKL pathway in ERBB2 (+) BC tumorigenesis. RANK signaling, probably through IKKα, has been reported to enhance spontaneous mammary tumorigenesis and metastatic potential in ERBB2-overexpressed BC models [10, 11]. Additionally, a selective pharmacological RANKL inhibitor could decrease tumor development and metastasis in ERBB2-positive context [10, 11]. Denosumab, a monoclonal antibody against RANKL approved for the treatment of osteoporosis and skeletal-related events [18], has been proposed to partially block the expansion of CSCs occurring in response to induced ERBB2 expression [15].

In this study, we examined the consistency and validity of our proposed theoretical model regarding the interplay between ERBB2 and RANK pathways in BC [19]. In this vein, we provide evidence, for the first time, of a physical interaction between RANK receptor and ERBB family members in BC cells. We show that RANK/ERBB2 dimer formation is related to ERBB2 expression and is disturbed by the presence of RANKL and ERBB2 inhibitors in ERBB2 (+) BC cells. The dimer disruption is accompanied by weakened NF-κB signaling and lower rates of cell proliferation and metastatic potential.

## Methods

### Antibodies and reagents

The following antibodies were used for Western blot (WB), immunohistochemistry (IHC), immunofluorescence (IF), and proximity ligation assay (PLA, Duolink). For WB: RANK antibody (H-300) (sc-9072), RANKL antibody (N-19) (sc-7628), IKKα antibody (3G12) (#11930), phospho-IKKα (phospho S176 + S180) antibody (ab17943), NF-κB p65 antibody (F-6) (sc-8008), phospho-NF-κB p65 (Ser536) antibody (93H1) (#3033), IκBα antibody (Η-4) (sc-1643), phospho-IκBα antibody (B-9) (sc-8404), anti-actin antibody, clone C4 MAB1501 (Millipore, MA). For IHC: RANK antibody (H-300) (sc-9072). For IF and PLA: RANK antibody (H-300) (sc-9072), RANKL antibody (N-19) (sc-7628), EGFR antibody (ab30), Neu antibody (3B5) (sc-33684), HER3 antibody (2F9) (ab91084), HER4 antibody (L20) (sc-31149). The secondary antibodies were employed: WB: goat anti-mouse IgG, HRPconjugate (12–349, Millipore), goat anti-rabbit IgG, HRPconjugate (12–348, Millipore). For IF: CF 543 Donkey Anti-Rabbit IgG (H + L), Highly Cross-Adsorbed (#20308, Biotinum). The following reagents were used in this study: recombinant human RANKL (390-TN-010, R&D Systems) dissolved in 0.1% BSA in PBS at 50 ng/ml, trastuzumab (Herceptin 150 mg, Roche) dissolved in a medium at 100 μg/ml, pertuzumab (Perjeta 420 mg, Roche) dissolved in a medium at 100 μg/ml, and denosumab (XGEVA 120 mg, AMGEN) dissolved in a medium at 100 μg/ml. The DUOLINK In Situ Ligation Kit was purchased from Sigma-Aldrich (DUO92002-Duolink In Situ PLA Probe Anti-Rabbit PLUS, DUO92004—Duolink In Situ PLA Probe Anti-Mouse MINUS, DUO92006—Duolink In Situ PLA Probe Anti-Goat MINUS, and DUO92007—Duolink In Situ Detection Reagents Orange). Nuclei were stained with NucBlue™ Fixed Cell ReadyProbes™ Reagent (R37606, Thermo Fisher Scientific), and coverslips were mounted with ProLong™ Diamond Antifade Mountant (P36965, Thermo Fisher Scientific).

### Cell culture and cell culture

SKBR3, MCF7, BT-474, and MDA-MB-453 cell lines are commonly used in breast cancer research. The cell lines were authenticated at the Laboratory of Genetics of the Biomedical Research Foundation of the Academy of Athens (Athens, Greece) by inverted DAPI banding karyotyping method. SKBR3 and BT-474 are characterized by ERBB2 overexpression. MCF7 and BT-474 are ER positive, and SKBR3 and MDA-MB-453 ER negative. The MCF10A human mammary epithelial cell line was used as normal cells. SKBR3 and MCF7 were cultured in DMEM, l-glutamine (Gibco, Life Technologies) and BT-474 and MDA-MB-453 in RPMI 1640 medium GlutaMAX (Gibco, Life Technologies), supplemented with 10% fetal bovine serum, FBS (Gibco, Life Technologies) and 1% penicillin-streptomycin. MCF10A cells were cultured in DMEM/F-12 (Gibco, Life Technologies) supplemented with 5% horse serum, 100 ng/ml cholera toxin, 20 ng/ml epidermal growth factor (EGF), 0.01 mg/ml insulin, 500 ng/ml hydrocortisone, and 1% penicillin-streptomycin. Cell cultures were incubated at 37 °C in a humidified atmosphere containing 5% CO_2_–95% air.

### Reverse transcription (RT)-PCR, semi-quantitative PCR

Total RNA was extracted from cultured cells using the RNeasy Mini Kit (Qiagen, CA, USA) according to the manufacturer’s instructions. cDNA was synthesized using the PrimeScript RT reagent kit-Perfect Real Time (Takara Bio, Japan) according to the manufacturer’s protocol.

For semi-quantitative PCR, the produced cDNA was amplified with specific primer pairs for—RANK-encoding—*TNFRSF11A* (annealing 60 °C, forward CCCGTTGCAGCTCAACAAG, reverse GCATTTGTCCGTGGAGGAA) and—RANKL-encoding—*TNFSF11* (annealing 60 °C, forward AGCAGAGAAAGCGATGGT, reverse GGGTATGAGAACTTGGGATT) genes (38 cycles) as well as with actin gene primer pairs (28 cycles) using KAPA 2G Multiplex Mastermix (KK5801, Sigma-Aldrich) according to the manufacturer’s instructions. PCR-amplified fragments were analyzed after their separation in agarose gels using image analysis software (ImageJ; La Jolla, CA) and normalized to actin gene levels.

### Western blot analysis

Protein extraction was performed using ice-cold RIPA buffer (Thermo Fisher Scientific). Bradford assay (Bio-Rad) was used to assess protein concentration in the extracts. Proteins were resolved by electrophoresis in SDS–polyacrylamide gels with several densities (10%, 12%, and 15%) depending on the molecular weight of each protein. Subsequently, they were transferred to a nitrocellulose membrane (Macherey–Nagel, Germany). Membranes were blocked for 1 h at room temperature in Tris-buffered saline with Tween-20 (TBS-T) with 5% nonfat milk. Then, membranes were incubated with primary antibodies overnight at 4OC (dilutions were 1:250 for antibodies against RANKL, IκBα, and p-IκBα; 1:500 for antibodies against p65 and RANK; 1:1000 for antibodies against IKKα, p-IKKα, and p-p65; and 1:2000 for antibody against actin). After incubation with HRP-conjugated secondary antibodies, the detection of the immunoreactive bands was performed with the Clarity Western ECL Substrate (Bio-Rad). Relative protein amounts were evaluated by a densitometry analysis using ImageJ software (La Jolla, CA, USA) and normalized to the corresponding actin levels.

### Cell proliferation assay

The assessment of breast cancer cell proliferation was performed with the XTT Cell Proliferation Assay Kit (10010200, Cayman Chemical, USA). Cells were seeded in a 96-well plate at a density of 10^3^–10^5^ cells/well in a culture medium. Cells were starved in phenol red-free medium supplemented with 5% charcoal stripped serum (CSS) for 24 h prior the treatments. Then, cells were cultured in a 100-μl starvation medium with or without the tested compounds in a CO_2_ incubator at 37 °C for variable time points. Afterwards, 10 μl of XTT Mixture was added to each well and mixed gently for 1 min on an orbital shaker. The cells were incubated for 2 h at 37 °C in a CO_2_ incubator. The absorbance of each sample was measured using a microplate reader at 450 nm.

### Migration assay

Breast cancer cells were seeded in 6-well plates and maintained in a CO_2_ incubator at 37 °C. The seeding density was adjusted appropriately for each cell line in order to form a confluent monolayer. The cell monolayer was scratched in a straight line with a sterile 200-μl pipet tip. The debris was removed by washing the cells once with PBS, and then it was replaced with a medium containing the tested compounds. The plates were placed under a phase-contrast, computer-assisted microscope, and the first image of the scratch was photographed at × 10 magnification. Reference points were made. The plates were placed in an incubator for 24 and 48 h. After completion of the incubation, plates were placed under a microscope, having reference points to align the photographed region, and images of the scratch were acquired. Images for each sample at 0, 24, and 48 h were analyzed quantitatively by using the TScratch software (Wimasis image analysis platform).

### Clonogenic assay

Breast cancer cells were seeded in 6-well plates, at an appropriate seeding density (~ 10^3^ cells/well). Cells were allowed to attach to the wells and then were treated. Plates were placed in a CO_2_ incubator at 37 °C for 10–15 days, until control cells formed sufficiently large colonies. Cells were then fixed with a solution containing 1 acetic acid:7 methanol and stained with 0.5% crystal violet in methanol for 15 min. Plates were carefully immersed in a tank with tap water and left to dry. Then, they were scanned, and the relative capacity to produce colonies was evaluated by a densitometry analysis using ImageJ software (La Jolla, CA, USA).

### Immunohistochemistry

The study included 20 archival BC tissue samples provided by the Department of Pathology, Medical School, National and Kapodistrian University of Athens. The tissue samples had been already evaluated for ERBB2 and ER expression. Immunohistochemistry was performed on FFPE sections cut at 5-μm thickness. Tissues were dried at 65 °C for 20 min, deparaffinized in xylene, and then rehydrated in an ethanol series. After washing with distilled H_2_O (dH_2_O), tissue sections were microwave heated with 10 mM citrate buffer (pH 6.0) for antigen retrieval for 25 min. To remove the endogenous peroxidase activity, sections were then treated with freshly prepared 3% hydrogen peroxide in methanol in the dark, for 10 min at room temperature. Non-specific antibody binding was blocked using 5% normal goat serum (NGS) for 1 h Sections were incubated with the primary antibody overnight at 4 °C (dilution was 1:100 for the antibody against RANK). Sections were then incubated at room temperature with biotinylated linking reagent (#20775, Merck, Darmstadt, Germany) for 10 min, followed by incubation with peroxidase-conjugated streptavidin label (#20774, Merck, Darmstadt, Germany) for 10 min. The sections were developed with diaminobenzidine and counterstained with hematoxylin (Sigma-Aldrich). Sections were dehydrated in ethanol buffers of 70, 80, 96, and 100% concentration and were mounted onto glass coverslips. PBS was used as the negative control instead of the primary antibody. Immunostaining was evaluated by the pathologist ST.

### Proximity ligation assay (PLA) and immunofluorescence (IF)

Duolink® Proximity Ligation Assay (PLA) allows in situ detection of protein-protein interactions. The PLA sensitivity is based on the fact that amplified signal is generated only when proteins of interest are in close proximity (< 40 nm). Breast cancer cells were cultured on 12-well chamber slides (81,201, ibidi). After reaching ~ 70% confluency, cells were starved for 24 h and treated accordingly for 1 h. Cells were fixed with 100% methanol for 5 min at − 20 °C.

For IF analysis, cells were blocked with 5% BSA in PBS for 1 h at room temperature, were stained for RANK (1:200) and RANKL (1:200) antibodies, and then, were incubated with fluorescent secondary antibodies (1:2000).

The PLA protocol was applied according to the manufacturer’s instructions (Sigma-Aldrich). Cells were blocked with Duolink® Blocking Solution and stained for RANK (1:200), EFGR (1:1000), HER2 (1:200), HER3 (1:500), and HER4 (1:200) antibodies. Cells were then incubated with the PLA probes diluted 1:5 in Duolink® Antibody Diluent. Subsequently, ligation and amplification steps were performed. All incubations were performed in a humidified chamber at 37 °C.

In both techniques, cells were counterstained with DAPI for nuclei staining and glass coverslips were mounted on the slides. Fluorescence images were acquired using a Leica TCS SP8 confocal microscope (Leica Microsystems, Wetzlar, Germany). At least ten random fields of view were selected and images were taken. Data analysis was performed using Duolink® Image Tool Software (Sigma-Aldrich), developed for objective quantification of PLA signals (Smal et al. 2010). Representative images for each condition are shown.

### Statistical analysis

All experiments were performed at least three times, and representative results of one experiment are shown. The data are presented as mean ± SD and analyzed by one-way ANOVA. GraphPad Prism 6 software was employed for these analyses. All statistical tests were two-sided. *p* values less than 0.05 were considered statistically significant.

## Results

### Detection of RANK and RANKL expression in human breast cancer cells and tissues

**Fig. 1:**
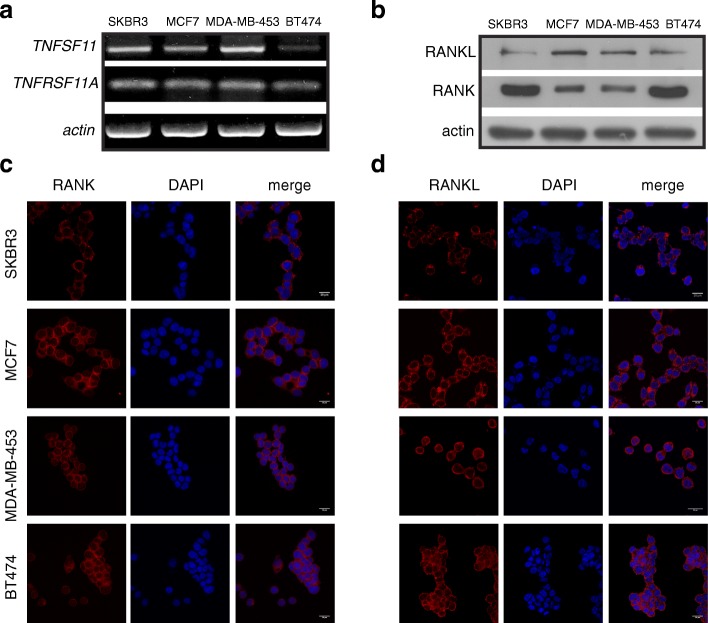
Detection of RANK and RANKL expression and subcellular localization in BC cells. **a** *TNFRSF11A* (RANK-encoding) and *TNFSF11* (RANKL-encoding) endogenous mRNA expression in SKBR3, MCF7, MDA-MB-453, and BT-474 BC cells. Data normalization was carried out against the *actin* housekeeping gene. **b** Western immunoblotting showing RANK and RANKL protein levels in human BC cell lines. Actin was used as a protein loading control. **c**, **d** The presence of RANK and RANKL was confirmed by immunofluorescence staining. Confocal images present also the subcellular localization of RANK and RANKL in BC cells. Nuclei were visualized using DAPI. Scale bars represent 20 μm. Representative images are shown

We first examined the expression levels of RANK receptor and its ligand, RANKL, in two ERBB2-positive, SKBR3 (ER-negative), BT-474 (ER-positive), and two ERBB2-negative MDA-MB-453 (ER-negative), MCF7 (ER-positive) BC cell lines. The endogenous protein and mRNA expression of RANK and RANKL was detected by Western immunoblotting and semi-quantitative PCR, respectively, in all four BC cell lines (Fig. 1a, b). Furthermore, using confocal microscopy, immunofluorescence staining of RANK and RANKL in BC cells confirmed the protein expression of RANK and RANKL and showed that the subcellular localization of RANK is in both cell membrane and cytoplasm (Fig. 1c). Concerning RANKL, it is localized mainly to cytoplasm (Fig. 1d). Finally, we examined RANK expression in a cohort of ERBB2+/ER+ (No 5), ERBB2+/ER− (No 5), ERBB 2−/ER+ (No 5), and TNBC (No 5) patients. A pathologist with no knowledge of the clinical data evaluated the immunohistochemical staining of the human paraffin-embedded BC tissues. RANK immunostaining was observed in all subsets of BC patients. Representative images with RANK-positive BC tissues are shown at Additional file 1: Figure S1.

### Physical association between RANK and ERBB family members

**Fig. 2:**
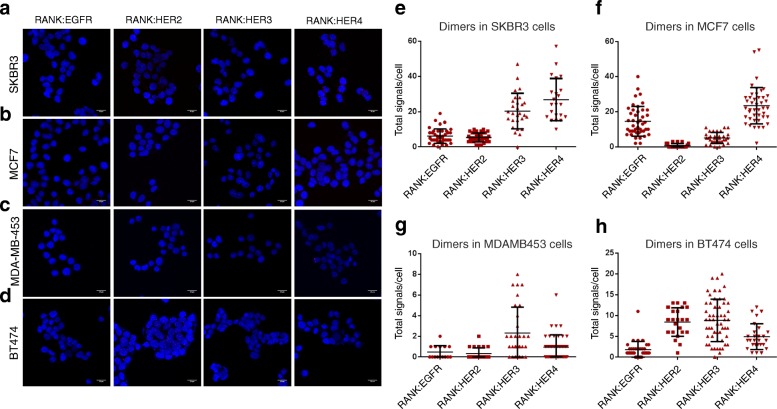
Dimer formation pattern between RANK receptor and ERBB family members in BC cells. **a**–**d** Representative images are shown using confocal microscopy. Red dots represent the positive signal of RANK-EGFR/ERBB2/ERBB3/ERBB4 heterodimers in SKBR3, MCF7, MDA-MB-453, and BT-474 cells using DUOLINK in situ proximity ligation assay (PLA). Scale bars represent 20 μm. **e**–**h** Dot plots represent the total signal per cell as analyzed by Duolink Image Tool Software. Data represent mean ± SD (standard deviation)

We examined the hypothesis that RANK could physically associate with ERBB family members. In order to find evidence for an association between RANK and ERBB family members, we performed proximity ligation assay (PLA) in the BC cells, SKBR3, MDA-MB-453, MCF7, and BT-474. As shown in Fig. 2, PLA signals are detected when RANK heterodimer formation with EGFR, ERBB2, ERBB3, and ERBB4 were tested, indicating that the receptors are physically associated in BC cells. In SKBR3 (PLA signal scales 0–60) and BT-474cells (PLA signal scales 0–25), there was a high incidence of RANK/ERBB2 heterodimers (5.4 and 8.2, respectively) (Fig. 2e, h). On the other hand, MCF7 (PLA signal scales 0–60) and MDA-MB-453 (PLA signal scales 0–10) present lower RANK/ERBB2 dimer number per cell, 0.7 and 0.3 respectively (Fig. 2f, g), indicating that there is a correlation between RANK/ERBB2 dimer formation and ERBB2 protein expression.

**Fig. 3:**
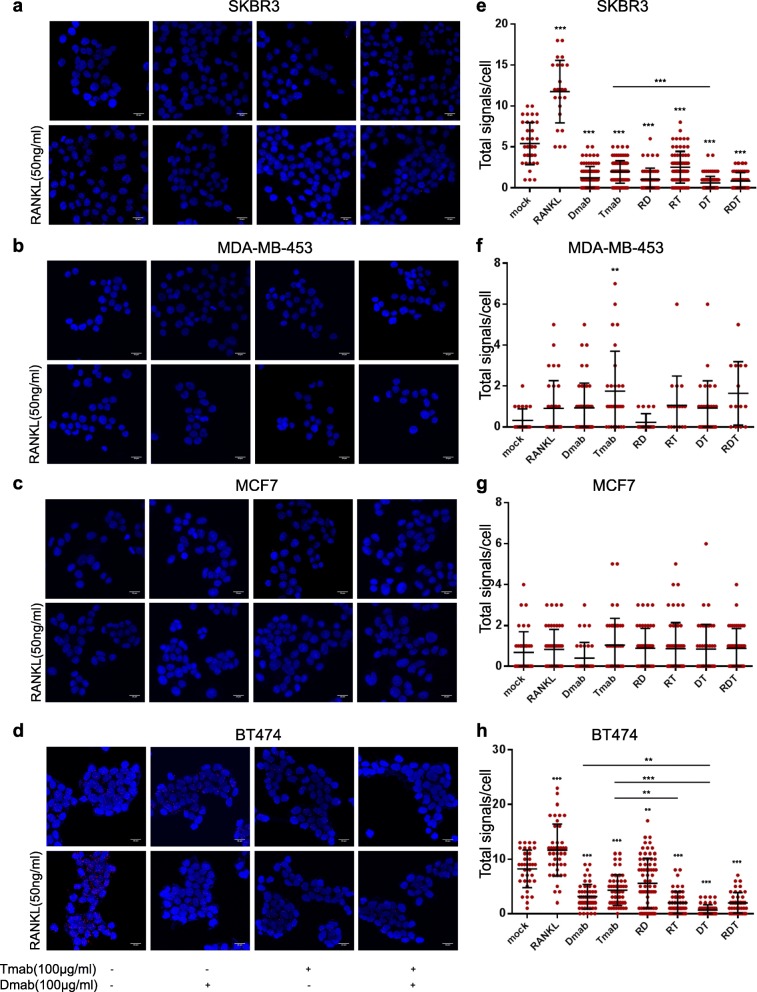
Dimer formation pattern between RANK and ERBB2 receptor in BC cells. **a**–**d** Representative images are shown using confocal microscopy. Red dots represent the positive signal of RANK/ERBB2 heterodimers in SKBR3, MCF7, MDA-MB-453, and BT-474 cells using DUOLINK in situ proximity ligation assay (PLA). Scale bars represent 20 μm. **e**–**h** Dot plots represent the total signal per cell as analyzed by Duolink Image Tool Software. Data represent mean ± SD. Asterisks indicate **p < 0.01 and ***p < 0.001 compared to mock cells for each panel

We subsequently investigated the formation of RANK/ERBB2 dimers with and without the addition of human sRANKL. In SKBR3 and BT-474 cells, we observed an increase of RANK/ERBB2 heterodimers compared to the control, 5.4 vs 11.8 and 8.2 vs 11.6, respectively (Fig. 3e, h). In MCF7 and MDA-MB-453, there was no significant alteration (Fig. 3f, g).

We went on to explore the formation of RANK/ERBB2 dimers with and without the addition of denosumab. Denosumab is a monoclonal antibody against RANKL, inhibiting the activation of RANK receptor. Denosumab has been recently approved for the treatment of osteoporosis and bone loss [18]. Notably, denosumab led to RANK/ERBB2 heterodimers’ decrease compared to the control cells in SKBR3 (1.2 vs 5.4) and BT-474 (3.1 vs 8.2), while no discernible difference was observed in MCF7 and MDA-MB-453 cells (Fig. 3). Moreover, the addition of RANKL to denosumab soared the formation of RANK/ERBB2 dimers compared to denosumab alone in BT-474 cells (5.6 vs 3.1) (Fig. 3h). Contrary to BT-474 cells, the co-treatment had no effect in SKBR3, MCF7, and MDA-MB-453 cells (Fig. 3e–g).

We next examined the effect of trastuzumab in the formation of RANK/ERBB2 dimers. Trastuzumab is a humanized monoclonal antibody targeting the extracellular domain IV of ERBB2 receptor and causes disruption of receptor dimerization [19]. In SKBR3 (1.9 vs 5.4) and BT-474 (4.3 vs 8.2) cells, the PLA signal was reduced in the presence of trastuzumab compared to mock cells (Fig. 3e, h). On the contrary, in MDA-MB-453 cells, there was a slightly high incidence of RANK/ERBB2 (0.3 vs 1.7) dimers (Fig. 3g), while MCF7 cells were not affected compared to control cells (Fig. 3f). Furthermore, the co-treatment of BT-474 cells with RANKL and trastuzumab compared to trastuzumab alone resulted in a lower percentage of RANK/ERBB2 (2.0 vs 4.3). On the other hand, the combination treatment of RANKL and trastuzumab had no effect on SKBR3, MCF7, and MDA-MB-453 cells.

We finally investigated the formation of RANK/ERBB2 dimers after dual inhibition with denosumab and trastuzumab. In SKBR3 cells, the combination treatment resulted in reduced PLA signal compared to mock and trastuzumab-treated cells (0.6 vs 5.4 vs 1.9) (Fig. 3e). In BT-474 cells, there was a reduction of RANK/ERBB2 heterodimer formation after dual inhibition compared with control, denosumab-treated, and trastuzumab-treated cells (0.7 vs 3.1 vs 4.3) (Fig. 3h). PLA signal did not exhibit any significant change in MCF7 and MDA-MB-453 cells. The addition of RANKL to denosumab plus trastuzumab combination had no further effect concerning the dimer formation in BC cells (Fig. 3).

PLA confirmed that RANKL has the ability to enhance the RANK/ERBB2 heterodimer formation, while denosumab and trastuzumab disrupt the dimers in ERBB2-positive BC cells. Our findings present evidence that combining trastuzumab with denosumab in ERBB2-positive BC cells diminishes ERBB2 dimerization with RANK more efficiently than single targeting.

### The impact of RANKL and ERBB2 inhibition in NF-κB signaling pathway downstream of RANK

We investigated the molecular significance of RANK/ERBB2 physical association in NF-κB signaling, the major downstream pathway of the RANK receptor. Specifically, we monitored the phosphorylation status of key components of canonical NF-κB pathway following RANK activation, RANKL and ERBB2 inhibition in BC cells. Based on concrete data from normal [20] and malignant mammary epithelia [16, 21] studies, highlighting the significance of IKK*α*, we focused on its modifications rather than IKK*β*.

**Fig. 4:**
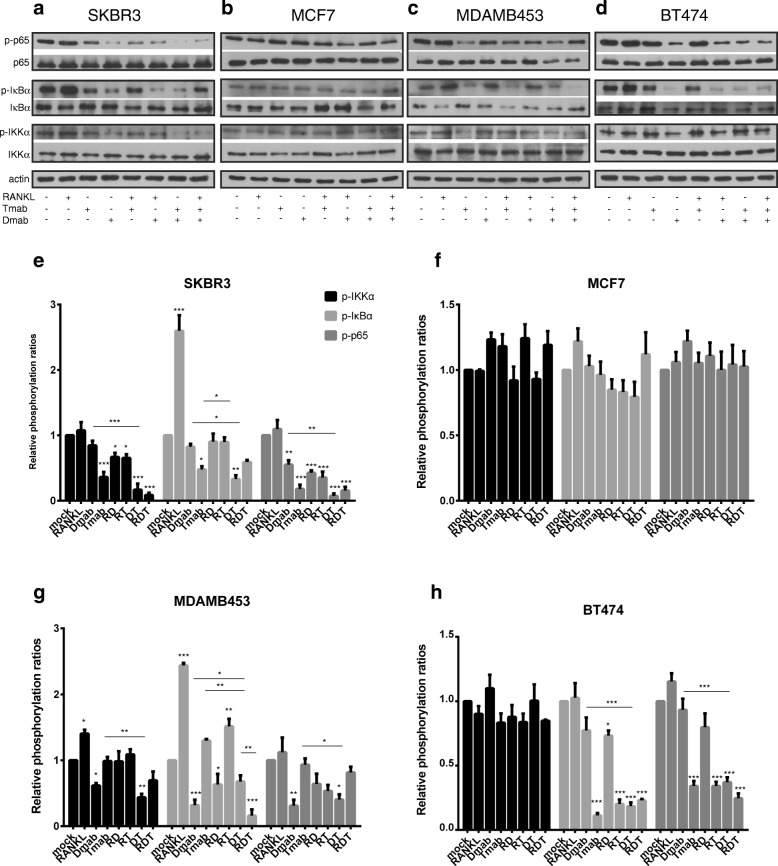
Effect of RANKL, denosumab, and trastuzumab in NF-κB signaling pathway in BC cells. **a**–**d** Cells were starved and treated with RANKL, denosumab, and/or trastuzumab. Western blot analysis from cell lysates of SKBR3, MCF7, MDA-MB-453, and BT-474 using phospho-IKK*α*, IKK*α*, phospho-IκB*α*, IκB*α*, phospho-NF-κB p65, and NF-κB p65 antibodies. Actin was used as a protein loading control. Representative Western blots of each cell line are presented. **e**–**h** The histograms represent densitometry results of the phospho-immunoblots normalized by total protein levels. Data were analyzed by one-way ANOVA and represent mean ± SD. Asterisks indicate *p < 0.05, **p < 0.01, and ***p < 0.001

Western blot analysis showed that RANKL treatment significantly increased phosphorylation of IκB*α*, the negative regulator of NF-κB transcriptional activity, compared to mock SKBR3 cells (Fig. 4a). Additional to phospho-IκB*α* elevation, MDA-MB-453 demonstrated increased phospho-IKK*α* levels (Fig. 4c). No significant change in activation of NF-κB pathway was observed in MCF7 and BT-474 after RANKL treatment (Fig. 4b, d).

To explore the effect of RANKL inhibition in NF-κB signaling, BC cells were treated with denosumab. The addition of denosumab in SKBR3 resulted in reduced phospho-p65 levels (Fig. 4e). MDA-MB-453 exhibited decreased phosphorylation of IKK*α*, IκB*α*, and NF-κB p65 compared to mock cells, following denosumab treatment **(**Fig. 4g). Denosumab does not seem to affect the NF-κB pathway in MCF7 and BT-474 (Fig. 4c, d). The addition of RANKL to denosumab in BC cells did not have a noteworthy effect in NF-κB pathway activation.

To verify the implication of ERBB2 in NF-κB pathway activation, we treated BC cells with trastuzumab. Comparing to the control, NF-κB pathway was significantly suppressed in SKBR3 cells (Fig. 4e), although the addition of RANKL seems to reverse this effect by increasing levels of phospho-IκB*α* compared to trastuzumab-treated SKBR3 cells (Fig. 4e). Trastuzumab treatment of BT-474 cells resulted in a dramatic reduction of IκB*α* and p65 phosphorylation and had no significant effect regarding IKK*α* activation (Fig. 4f). As expected, MCF7 and MDA-MB-453 did not correspond to the inhibitor (Fig. 4g, h).

We finally investigated the effect of RANKL and ERBB2 dual inhibition. The combination treatment showed a more suppressive effect than denosumab alone in SKBR3 cells (Fig. 4e). The addition of RANKL slightly increased the phosphorylation of IκB*α* and NF-κB p65 compared to the dual inhibited cells (Fig. 4e). MDA-MB-453 dual treated cells exhibited lower phosphorylation levels of IKK*α* and p65 in relation to control cells, while denosumab efficiency regarding reduction of phospho-IκB*α* is weaken when trastuzumab is added (Fig. 4g). On the other hand, the combination treatment did not offer any advantage to BT-474 cells compared to trastuzumab (Fig. 4h). MCF7 cells did not exhibit any significant change (Fig. 4f).

These data suggest that RANKL and denosumab have the ability to affect NF-κB signaling in SKBR3 and MDA-MB-453 cells. Worth mentioning, the RANKL stimulation minimized the efficacy of trastuzumab in SKBR3. On the contrary, denosumab addition to trastuzumab enhanced the suppressive action of both inhibitors.

### Denosumab and trastuzumab suppress aggressive properties of SKBR3 cells in vitro

**Fig. 5:**
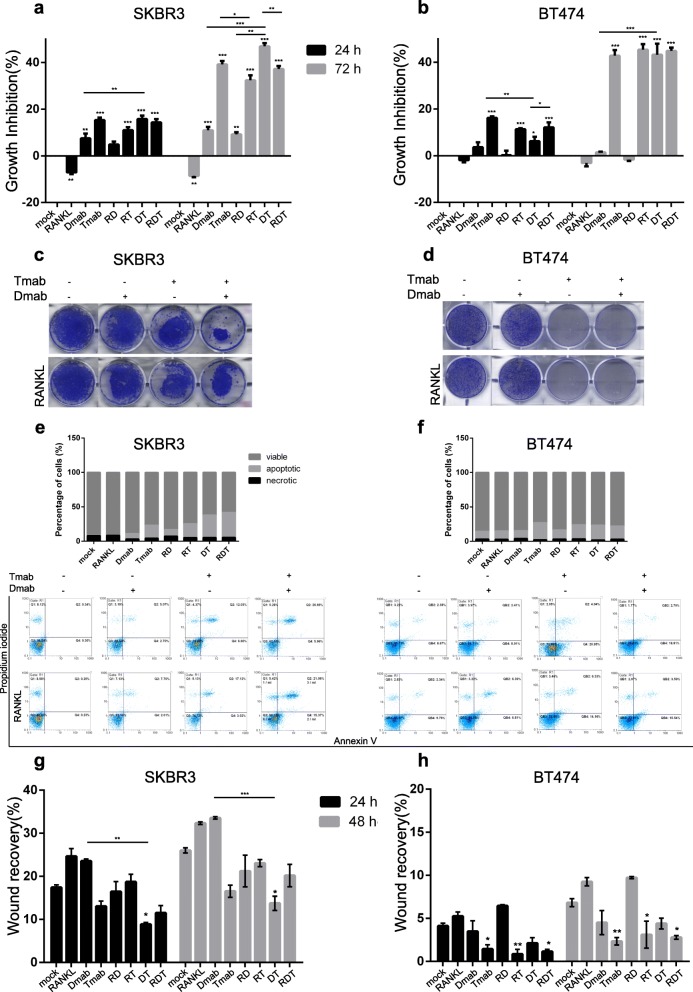
Effect of denosumab and trastuzumab in cell properties of breast cancer cells. **a**, **b** XTT proliferation assay for SKBR3 and BT-474 cells, after treatment with RANKL, denosumab, and/or trastuzumab for 24 and 72 h. Results are expressed in the histogram as growth inhibition, normalized to the control group. **c**, **d** Representative images of clonogenic assay performed at SKBR3 and BT-474 cells. **e**, **f** The histograms represent data from FACS analysis using annexin V-FITC and propidium iodide (PI) of SKBR3 and BT-474 cells after 48 h of treatment. The histograms show the distribution of cells as percentage of viable, apoptotic and necrotic. **g**, **h** Quantification of migration-wound healing assay for SKBR3 and BT-474 cells analyzed at 24 and 48 h. The histogram shows percent wound recovery at 24 and 48 h in relevance to 0 h. Data in **a**–**h** were analyzed by one-way ANOVA and represent mean ± SD. Asterisks indicate *p < 0.05, **p < 0.01, and ***p < 0.001

Next, we performed in vitro functional analyses in order to assess if the observed downregulation of NF-κB signaling (Fig. 4) following denosumab and trastuzumab treatment had an impact in BC cell properties. Prior to the evaluation of the effect of denosumab and trastuzumab on the viability of BC cells, XTT assay was performed to assess the toxicity of the inhibitors in normal MCF10A cells (Additional file 2: Figure S2). In the presence of RANKL, SKBR3 (Fig. 5a), and MDA-MB-453 (Additional file 3: Figure S3), cells exhibited significantly increased cell proliferation on 24 and 72 h compared to the control group. In contrast, denosumab inhibited cell growth in these cell lines. As expected, trastuzumab-treated SKBR3 and BT-474 displayed reduced cell proliferation (Fig. 5a, b). Interestingly, the addition of RANKL to trastuzumab led to the recovery of SKBR3 cell growth on 72 h (*p* < 0.05). Combination treatment of SKBR3 cells with denosumab and trastuzumab resulted in a more effective growth inhibition compared to single targeting on 72 h (D vs DT, *p* < 0.001, T vs DT, *p* < 0.01). However, the addition of RANKL reversed this effect (DT vs RDT, *p* < 0.01). The superiority of dual inhibition was confirmed by clonogenic assay in SKBR3 cells (Fig. 5c). Interestingly, the clonogenic survival of SKBR3 cells was affected by the addition of RANKL to denosumab plus trastuzumab (Fig. 5c). Moreover, trastuzumab dramatically reduced the capacity of BT-474 cells to form colonies (Fig. 5d).

Subsequently, we conducted flow cytometry analysis of annexin V and propidium iodide (PI) staining in ERBB2 (+) BC cells, in order to further examine the effect of dual inhibition in programmed cell death. The assay results indicate that 48-h treatment with denosumab or trastuzumab induced apoptosis in SKBR3 cells, while dual inhibition seems to be more effective compared to single targeting (Fig. 5e). In contrast to proliferation data, the addition of RANKL had no significant effect on SKBR3 cell death. Although trastuzumab significantly induced apoptosis in BT-474 cells, RANKL and denosumab had a minor effect on cell distribution compared to the control group (Fig. 5f).

Furthermore, we examined the effect of denosumab and trastuzumab on metastatic cell capacity of BC cells. Combination treatment for 24 and 48 h efficiently repressed motility in wound healing migration assays compared to mock (*p* < 0.05) and denosumab-treated (*p* < 0.01 and *p* < 0.001 respectively) SKBR3 cells (Fig. 5g). BT-474 metastatic potential is affected mainly by the presence of trastuzumab, either alone (*p* < 0.05 referred to 24 h, *p* < 0.01 referred to 48 h), with RANKL (*p* < 0.01 referred to 24 h, *p* < 0.05 referred to 48 h), or with RANKL plus denosumab (*p* < 0.05) (Fig. 5h). Regarding MCF7 and MDA-MB-453 cells, the combination treatment with RANKL, denosumab, and trastuzumab significantly reduced the wound recovery rate after 24 (*p* < 0.05) and 48 h (*p* < 0.05) respectively (Additional file 3: Figure S3).

Data from proliferation, colony formation, cell death, and migration assays performed at BC cells (Fig. 5) indicate that the addition of denosumab to trastuzumab treatment strengthens the anti-proliferative and anti-migrative effect in ERBB2-positive, ER-negative cells.

### Denosumab’s anti-tumor effect is enhanced by dual ERBB2 blockage in SKBR3 cells

**Fig. 6:**
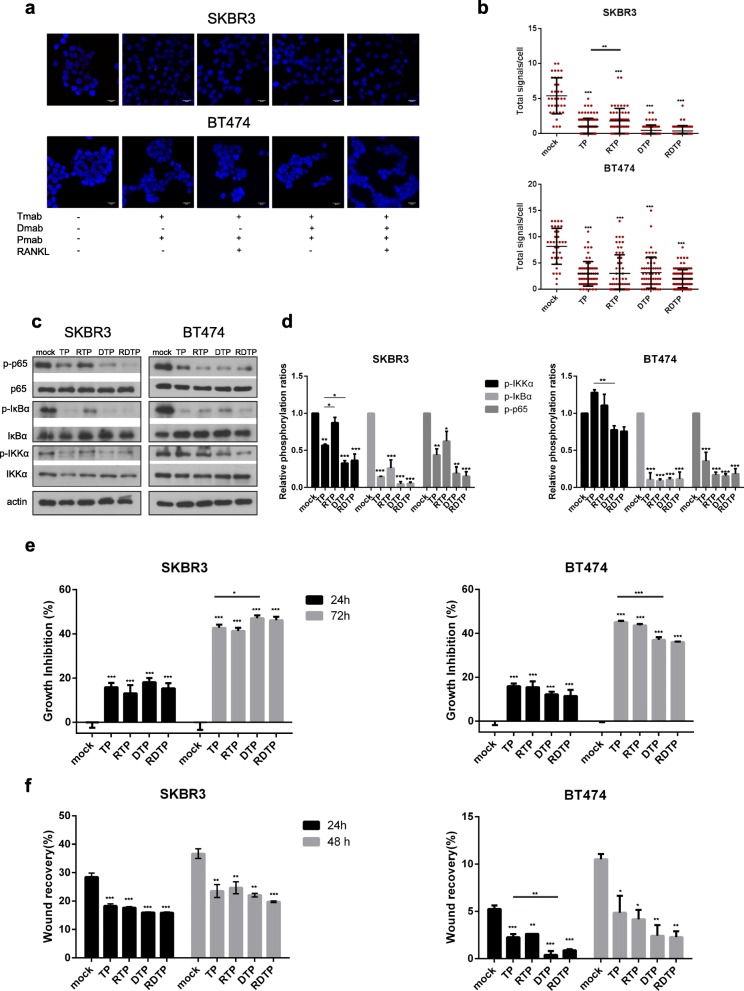
Effect of pertuzumab addition to denosumab plus trastuzumab in ERBB2-positive BC cells. Cells were starved and treated as indicated. **a** Representative images are shown using confocal microscopy. Red dots represent the positive signal of RANK/ERBB2 heterodimers in SKBR3 and BT-474 cells using DUOLINK in situ proximity ligation assay (PLA). Scale bars represent 20 μm. **b** Dot plots represent the total signal per cell as analyzed by Duolink Image Tool Software. **c** Western blot analysis from cell lysates of SKBR3 and BT-474 using phospho-IKK*α*, IKK*α*, phospho-IκB*α*, IκB*α*, phospho-NF-κB p65, and NF-κB p65 antibodies. Actin was used as a protein loading control. Representative Western blots of each cell line are presented. **d** The histograms represent densitometry results of the phospho-immunoblots normalized by total protein levels. **e** XTT proliferation assay for SKBR3 and BT-474 cells, after 24 and 72 h of treatment. Results are expressed in the histogram as growth inhibition, normalized to the control group. **f** Quantification of migration-wound healing assay for SKBR3 and BT-474 cells analyzed at 24 and 48 h. The histogram shows percent wound recovery at 24 and 48 h in relevance to 0 h. Data in **b**–**f** were analyzed by one-way ANOVA and represent mean ± SD. Asterisks indicate *p < 0.05, **p < 0.01, and ***p < 0.001

Nowadays, trastuzumab- and pertuzumab-based therapy is considered as a typical strategy in clinical setting regarding ERBB2-positive BC patients [22]. Thus, we examined the effects of pertuzumab addition in denosumab plus trastuzumab treatment in ERBB2 (+) BC cells. SKBR3 and BT-474 cells were treated, and Duolink assay was performed (Fig. 6a). Following combination treatment with pertuzumab and trastuzumab, RANK/ERBB2 dimers were further decreased in comparison with trastuzumab-treated SKBR3 cells (PLA signal scales 0–15), while RANKL addition to this regiment restricted the aforementioned reduction (Fig. 6b). In addition, the triple-drug treatment resulted in a significant lower number of dimers compared to denosumab, trastuzumab, and trastuzumab-pertuzumab-treated SKBR3 cells (Fig. 6b). On the contrary, pertuzumab addition to any combination had no additional effect in dimer formation in BT-474 cells (PLA signal scales 0–20) (Fig. 6b).

Subsequently, we evaluated whether pertuzumab addition offers an advantage to downregulation of NF-κB signaling. Specifically, western blot analysis revealed that, following trastuzumab-pertuzumab treatment, phosphorylation of IKK*α*, IκB*α*, and NF-κB p65 was significantly decreased compared to mock SKBR3 cells, and there was a slight increase when RANKL was added (Fig. 6d). Moreover, triple-drug treatment had an additional effect regarding IKK*α* phosphorylation reduction when compared with trastuzumab-pertuzumab-treated SKBR3 cells (Fig. 6d). As far BT-474 cells, all combination treatments affected NF-κB signaling by reducing phospho-IκB*α* and phospho-p65 (Fig. 6d). However, IKK*α* phosphorylation seemed unaffected after trastuzumab, pertuzumab, and/or denosumab treatment.

Finally, we assessed the toxicity of denosumab and trastuzumab plus pertuzumab in normal MCF10A cells (Additional file 2: Figure S2), and subsequently, we evaluated the anti-tumor effect of the inhibitors, performing XTT and wound healing assays in BC cells. Following 72-h triple-drug treatment, proliferation rate was significantly lower than trastuzumab-pertuzumab-treated SKBR3 cells (*p* < 0.05) (Fig. 6e). Treatment with trastuzumab, pertuzumab, and/or denosumab dramatically decreased survival in relation to mock BT-474 cells (Fig. 6e). RANKL addition did not affect the cell growth of neither SKBR3 nor BT-474 (Fig. 6e). Data from scratch assays revealed the efficient inhibition of migration rate after triple targeting, which was unaffected in the presence of RANKL in SKBR3 and BT-474 cells (Fig. 6f).

Taken together these findings, pertuzumab addition to denosumab plus trastuzumab treatment offers an advantage regarding RANKL effect on trastuzumab and pertuzumab efficacy in SKBR3 cells.

## Discussion

RANK and RANKL are expressed on various cell types and through their signaling can modulate vital cellular functions. Among the cellular systems that RANK axis has been reported to be implicated is mammary tumorigenesis. Interestingly, there are scientific reports revealing the involvement of RANK signaling in each stage of breast carcinogenesis [10–12, 14]. Concrete data support RANK role in hormone [10, 13] and *BRCA1* [14]-associated BCs. Additionally, an implication in ERBB2-positive breast carcinomas is indicated [10, 11, 21]. In this study, we explored and revealed a novel role of RANK as physical partner of ERBB family members in RANK-expressing BC cells, capable to modulate NF-κB signaling pathway resulting in the regulation of proliferation and survival of ERBB2-positive, ER-negative BC cells.

In vitro experiments in differentiating osteoclasts revealed for the first time a functional and physical association between RANK and EGFR signaling [23]. Particularly, RANKL stimulation promotes EGFR expression and transactivation. Subsequently, activated EGFR forms a multiprotein complex including RANK, which leads to enhanced RANK downstream signaling, regulating osteoclast differentiation and survival [23]. Of note, similar results have been observed in primary BC [24]. They provided preliminary evidence of RANK interaction with EGFR pathways. A positive correlation between RANK (*TNFRSF11A*) and *EGFR* expression in BC was indicated [24]. Moreover, RANK and EGFR co-expression is characterized by worse clinical outcome, enhanced downstream pathways, and induced cellular invasiveness in BC cells [24]. Our findings from proximity ligation assay is an extension of the aforementioned studies revealing for the first time a direct association of RANK with ERBB family members (EGFR/ERBB2/ERBB3/ERBB4) in BC cells.

Dimer formation between RANK and ERBB2 seemed to be positively related to ERBB2 expression; thus, SKBR3 and BT-474, ERBB2-positive cells, exhibit a high number of RANK/ERBB2 dimers. RANKL treatment resulted in increased RANK/ERBB2 dimerization, while treatment with denosumab, trastuzumab, and/or pertuzumab had the opposite effect in ERBB2 (+) BC cells. Our findings are in accordance with previous studies in osteoclasts showing that RANK and EGFR association was enhanced by osteoclast differentiation medium, containing RANKL, and reduced by AG1478, a EGFR kinase inhibitor, treatment [23]. RANKL stimulation seems to be crucial as its addition and inhibition affect RANK/ERBB2 dimerization pattern. The observation that dual targeting with trastuzumab and pertuzumab is more effective compared to trastuzumab, combined with the inhibitors molecular action [25], leads to the assumption that ERBB2 associates with RANK as part of active homodimer or heterodimer with other members of ERBB family, forming a multiprotein complex.

Previous studies in osteoclasts and BC cells revealed that EGFR is implicated in augmentation of RANK downstream signaling [23, 24]. NF-κB signaling is the main RANK downstream pathway. Activated NF-κB has been associated with tumor growth [16] and drug resistance [26] in ERBB2-positive breast cancer. NF-κB is activated through canonical and non-canonical signaling pathways. Although IKK*β* is considered to be a key mediator in the canonical pathway, data from normal [20] and malignant [21] mammary revealed also a role of IKK*α* in canonical pathway. IKK*α* is necessary for ERBB2-induced breast tumorigenesis and plays an essential role in self-renewal of cancer stem cells [16]. RANKL upregulated the phosphorylation and nuclear translocation of IKK*α* in a ERBB2-induced mammary carcinoma cell line [11]. Additionally, RANKL stimulation induced NF-κB activation in SKBR3 cells [13]. In line with that, we observed a significant increase of phospho-IκB*α*; however, IKK*α* and p65 phosphorylation were slightly increased following treatment of SKBR3 with RANKL. Moreover, studies reported that lapatinib and trastuzumab decreased NF-κB signaling in SKBR3 cells [21, 27]. Of note, additional to reduced phosphorylation levels of IκB*α* and p65, we noticed a significant downregulation of phospho-IKK*α* in SKBR3-treated cells with trastuzumab and trastuzumab plus pertuzumab. Merkhofer and colleagues, in an attempt to elucidate the pathway leading to NF-κB activation, proved that ERBB2 requires IKK*α* to activate NF-κB through the canonical pathway independently of the PI3K pathway in ERBB2-positive breast cancer cells [21]. Thus, ERBB2 might hold a regulatory role in IKK*α*-mediated canonical pathway. Interestingly, the addition of RANKL to anti-ERBB2 drugs seemed to decrease inhibitors’ efficacy. Whereas when denosumab was added to the combination, RANKL had no further effect on downregulation of NF-κB signaling in SKBR3 cells. This data suggests that ERBB2 cooperate with RANK to regulate IKK*α*-mediated canonical signaling.

ERBB2 activation of NF-κB via IKK*α* promotes invasive phenotype in ERBB2-positive BC cells [21]. Particularly, NF-κB promotes proliferation and survival, inducing ERBB2-mediated mammary tumorigenesis [26]. RANK signaling stimulates proliferation and survival of breast tumor cells through induction of cyclin D1 and Bcl-2 protein expression [28]. IKK*α* plays a crucial role in NF-κB-cyclin D1-mediated proliferation of cancer cells [13]. Accordingly, data from proliferation, apoptosis, and clonogenic assay revealed that RANKL stimulation enhanced proliferation, while survival was quite unaffected in SKBR3 cells. Interestingly, SKBR3 cells exhibited decreased rates of proliferation and enhanced apoptosis when treated with a combination of denosumab with trastuzumab. RANKL addition to the combination diminished the efficacy of the inhibitors. However, denosumab plus dual ERBB2 targeting seemed to be more effective concerning growth inhibition and tolerance to RANK ligand.

RANKL promotes migration, invasion, and metastasis, by activation of NF-κB signaling and subsequent upregulation of Snail and Twist, key regulators of epithelial to mesenchymal transition (EMT) [29]. RANKL signaling has, also, a vital role in the expansion of BC stem cells (CSCs) [13], leading probably to a robust metastatic activity. RANKL has been demonstrated to mediate CSC self-renewal in ERBB2-positive cells [15]. Notably, IKK*α* is essential to this process [16]. In particular, IKK*α* has been shown to regulate ERBB2-induced CSC expansion by stimulating the nuclear export of p27, a negative regulator of the G1–S transition [30]. Our observations about RANKL treatment having no additional effect on migration capacity are in contrast with the aforementioned studies. However, denosumab combined with trastuzumab and/or pertuzumab significantly decreased migration rate in SKBR3 cells. This is in agreement with previous findings showing that RANKL inhibition decreased proliferation and metastases in ERBB2 transgenic mice [10]. In this line, RANK+/− ERBB2 transgenic mice exhibited reduced metastatic rates compared with homozygotes [11]. Furthermore, silencing or blocking RANK diminished metastatic rate, while RANKL treatment had the opposite effect in ERBB2-induced mammary carcinomas [11].

Based on the data described so far, we can assume the importance of RANK signaling in proliferation, survival and metastatic potential of ERBB2 positive BC cells. Also, it is reasonable to conclude that combinatorial targeting of ERBB2 and RANKL could be a more effective approach, which would overcome RANKL effect in suppressing anti-tumor action of anti-ERBB2 agents. There are evidence supporting that NF-κB signaling might also constitute an essential mechanism of resistance to anti-ERBB2 strategies when applied for the BC treatment [26, 31]. Following this rationale, the combination of anti-ERBB2 agents with proteasome inhibitors (which block IκB degradation and thus NF-κB activation) [31] and NF-κB inhibitors [26, 32] has been successfully tested as a novel therapeutic strategy for treating ERBB2-positive BC patients.

RANK expression has been linked with hormone receptor negativity, high pathological grade, and worse clinical outcome [33]. Moreover, a recently published study revealed an association of RANK and RANKL dual expression with poor clinical outcomes in triple-negative BC (TNBC) [34]. Interestingly, our research using MDA-MB-453 cells demonstrated that RANKL stimulation and inhibition affected NF-κB signaling, proliferation, and slightly migration, indicating a RANK role in TNBC.

## Conclusions

In conclusion, this study reveals for the first time a functional and physical association between RANK receptor and ERBB family members. RANK dimerization with ERBB2 seems to play a fundamental role in the progress of ERBB2-positive breast carcinomas. We therefore propose a novel molecular mechanism concerning a regulatory role of ERBB2, mediated through functional cross-talk with RANK, in NF-κB signaling, a key RANK downstream pathway. IKK*α* is mainly responsible for NF-κB canonical pathway activation, required for breast tumorigenesis and drug resistance. The noticeable anti-tumor effect at molecular and cellular level of denosumab, when combined with anti-ERBB2 agents in ERBB2-positive BC cells, raises the possibility for therapeutic strategies with existing drugs, at least in a specific BC subgroup of RANK-expressing ERBB2-positive patients. Our in vitro findings strongly encourage further preclinical investigations to be conducted and highlight the need of setting up clinical trials to evaluate whether double pathway inhibition strategy in the double-positive cohort of breast cancer patients would lead to significant advantages.

## Supplementary information

**Additional file 1: Figure S1.** Immunohistochemical expression patterns of RANK in BC patients (× 40). **(a)** RANK expression in ERBB2 positive BC tissue. **(b)** Expression of RANK in ERBB2 negative/ ER positive cancerous breast tissue. **(c)** RANK protein expression in a TNBC patient sample. **(d)** RANK protein expression in an ERBB2 positive/ ER positive BC patient sample. Scale bars represent 50 μm.

**Additional file 2: Figure S2. (a)** XTT proliferation assay for MCF10A cells, after 24 and 72 h treatment, in order to evaluate denosumab and trastuzumab toxicity. **(b)** XTT proliferation assay for MCF10A cells, after 24 and 72 h treatment, in order to evaluate the toxicity of denosumab, trastuzumab and pertuzumab. Results in **a** and **b** are expressed in the histogram as growth inhibition, normalized to the control group. Data in **a** and **b**, were analyzed by one-way ANOVA and represent mean ± SD.

**Additional file 3: Figure S3. (a)** XTT proliferation assay for MCF7 and MDA-MB-453 cells, after treatment with RANKL, denosumab and/ or trastuzumab for 24 and 72 h. Results are expressed in the histogram as growth inhibition, normalized to the control group. **(b)** Quantification of migration- wound healing assay for MCF7 and MDA-MB-453 cells analyzed at 24 and 48 h. The histogram shows percent wound recovery at 24 and 48 h in relevance to 0 h. Data in **a** and **b**, were analyzed by one-way ANOVA and represent mean ± SD. Asterisks indicate *p < 0.05, **p < 0.01, ***p < 0.001.

## Data Availability

All data generated or analyzed during this study are included in this manuscript (and its supplementary file). The datasets used and/or analyzed during this study are available from the corresponding author at a reasonable request.

## Abbreviations

BC: Breast cancer
BRCA1: Breast cancer susceptibility gene 1
CSC: Cancer stem cell
EGFR: Epidermal growth factor receptor
EMT: Epithelial to mesenchymal transition
ERBB2: Human epidermal growth factor receptor 2
ER-α: Estrogen receptor-α
IF: Immunofluorescence
IKK: Inhibitor of NF-κB kinase
IκB: Inhibitor of NF-κB
NF-κB: Nuclear factor-κB
PI3K: Phosphatidyl inositol 3-kinase
PLA: Proximity ligation assay
PR: Progesterone receptor
RANK: Receptor activator of NF-κB
RANKL: RANK ligand
RTK: Receptor tyrosine kinase
RT-PCR: Reverse transcription-PCR (polymerase chain reaction)
WB: Western blot
