# Supplementary figures and images for: Combining RANK/RANKL and ERBB-2 targeting as a novel strategy in ERBB-2-positive breast carcinomas
Source: Breast Cancer Res. 2019 Dec 3;21:132. doi: 10.1186/s13058-019-1226-9 (PMC6892243; doi:10.1186/s13058-019-1226-9)

**a**

ERBB2+/ ER-

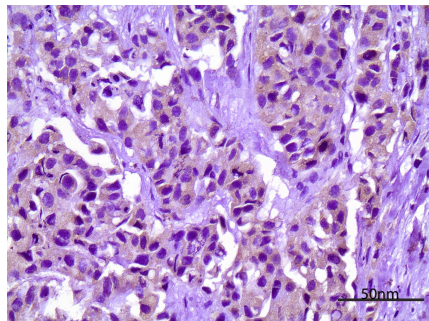**b**

ERBB2-/ ER+

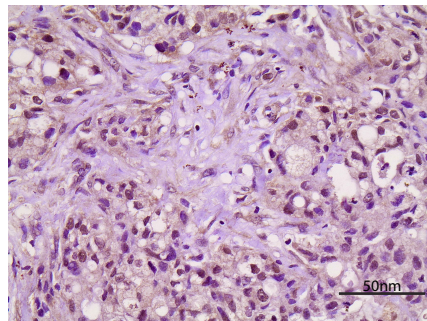**c**

ERBB2-/ ER-

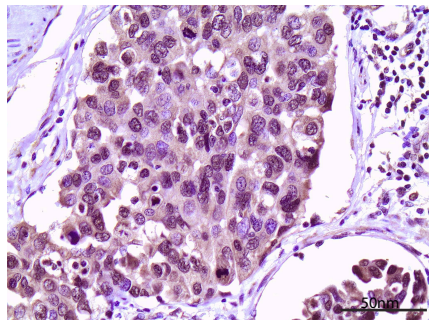**d**

ERBB2+/ ER+

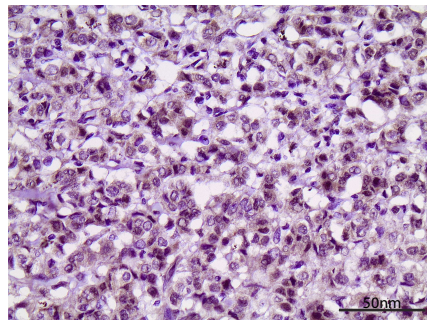

Supplement: Supplementary file 1 — Additional file 1: Figure S1. Immunohistochemical expression patterns of RANK in BC patients (× 40). (a) RANK expression in ERBB2 positive BC tissue. (b) Expression of RANK in ERBB2 negative/ ER positive cancerous breast tissue. (c) RANK protein expression in a TNBC patient sample. (d) RANK protein expression in an ERBB2 positive/ ER positive BC patient sample. Scale bars represent 50 μm. [file 13058_2019_1226_MOESM1_ESM.pdf]

**a****MCF10A**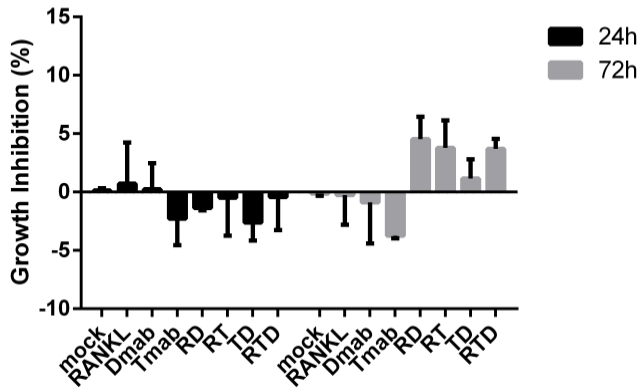**b****MCF10A**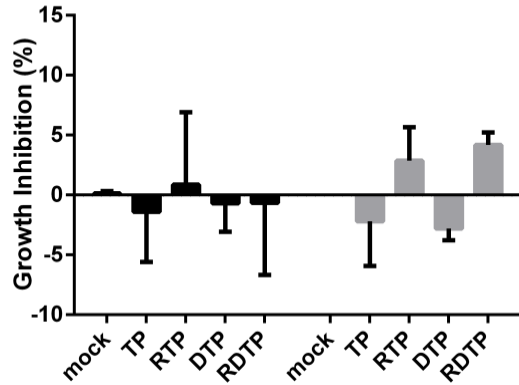

Supplement: Supplementary file 2 — Additional file 2: Figure S2. (a) XTT proliferation assay for MCF10A cells, after 24 and 72 h treatment, in order to evaluate denosumab and trastuzumab toxicity. (b) XTT proliferation assay for MCF10A cells, after 24 and 72 h treatment, in order to evaluate the toxicity of denosumab, trastuzumab and pertuzumab. Results in a and b are expressed in the histogram as growth inhibition, normalized to the control group. Data in a and b, were analyzed by one-way ANOVA and represent mean ± SD. [file 13058_2019_1226_MOESM2_ESM.pdf]

**a**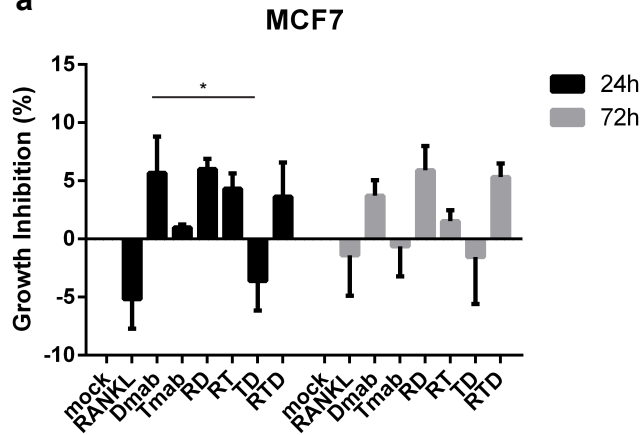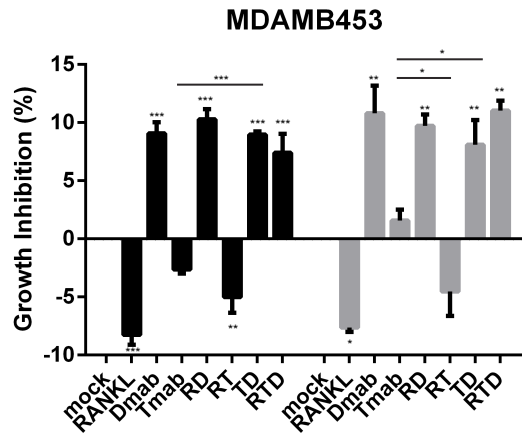**b**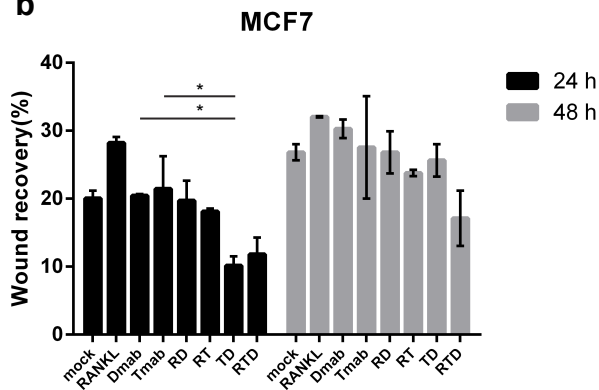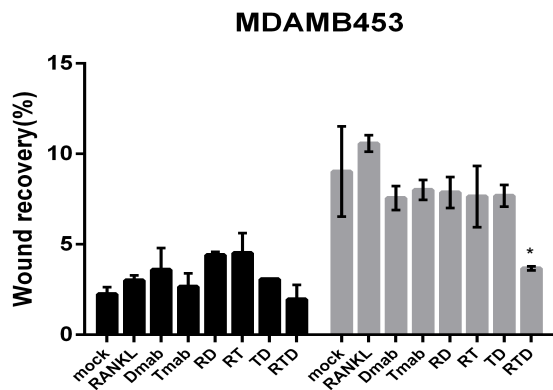

Supplement: Supplementary file 3 — Additional file 3: Figure S3. (a) XTT proliferation assay for MCF7 and MDA-MB-453 cells, after treatment with RANKL, denosumab and/ or trastuzumab for 24 and 72 h. Results are expressed in the histogram as growth inhibition, normalized to the control group. (b) Quantification of migration- wound healing assay for MCF7 and MDA-MB-453 cells analyzed at 24 and 48 h. The histogram shows percent wound recovery at 24 and 48 h in relevance to 0 h. Data in a and b, were analyzed by one-way ANOVA and represent mean ± SD. Asterisks indicate *p < 0.05, **p < 0.01, ***p < 0.001. [file 13058_2019_1226_MOESM3_ESM.pdf]
